# Surgical treatment of patellar tendon rupture after total knee arthroplasty with a double-row repair method using the hamstring tendons: A novel technique with functional results

**DOI:** 10.1097/MD.0000000000037875

**Published:** 2024-04-26

**Authors:** Erkan Akgun, Abdulsamet Emet, Emre Tepedelenlioglu, Kemal Sibar, Halil Gok, Ahmet Firat

**Affiliations:** a Department of Orthopedics and Traumatology, Etlik City Hospital, Ankara, Turkey.

**Keywords:** double-row reconstruction, hamstring tendon autograft, patellar tendon rupture, total knee arthroplasty

## Abstract

**Background:**

Patellar tendon rupture (PTR) is extremely rare but serious complication after primary or revision total knee arthroplasty. Due to the serious failure rates of end-to-end repair techniques, various augmentation techniques have been described. In this study, the results of patients with PTR after reconstruction using our own technique with semitendinosus (ST) and gracilis tendons taken from the affected side were evaluated retrospectively.

**Methods:**

A total of 14 patients, whose diagnosis was made based on physical examination and clinical findings, and supported radiologically (ultrasonography), were included in the study. In these patients, reconstruction was performed using double-row repair technique with the ST and gracilis tendons. Active-passive knee joint range of motion, active knee extension loss, and the Caton-Deschamps index at preoperative and final follow-up visits were compared. Tegner-Lysholm knee score and Kujala score were used to evaluate functional results.

**Results:**

In 14 patients (8 women and 6 men) with a mean age of 68.1 years, the median time between injury and surgery was 6.6 weeks. In all patients, the rupture was in the distal part of the patellar tendon. While the median preoperative Caton-Deschamps index was 1.8, the postoperative median value was found to be 1.25 after an average follow-up of 3.8 years (*P* = .014). The median preoperative knee extension loss decreased from 25° to 5° postoperatively. Tegner-Lysholm knee score and Kujala score of the patients at their last follow-up were significantly increased (*P* < .01).

**Conclusion:**

For PTR developing after total knee arthroplasty, the double-row reconstruction technique with ST and gracilis tendons is effective.

## 1. Introduction

Extensor mechanism injuries are rare and the incidence after total knee arthroplasty (TKA) varies between 0.17% and 2.5%, but they are a serious complication after primary or revision TKA. In the literature, it is seen at a higher rate after TKA revision.^[1–3]^ Tears usually occur in the tibial attachment of the patellar tendon and may occur intraoperatively, early postoperatively, or late period. Although poor surgical procedures, inappropriate prosthesis design and excessive physical therapy protocols are shown to be among the most important causes of early-stage tears, its etiology is complex and multifactorial.^[4,5]^

In patients who develop patellar tendon rupture (PTR) after TKA surgery, extension failure causes instability and uncontrolled falling, accelerating implant failure.^[6]^ Many different techniques have been described in the management of the treatment of this complication, but common treatment strategy has not been established. Primary tendon repair can be applied in the acute period and in partial tears, but due to the high failure rates, especially in full-thickness tears exceeding 2 weeks, augmentation with various biological or artificial tendon grafts is recommended.^[7–12]^ Autogenous hamstring tendons, allografts containing bone tissue and artificial tendon grafts are the most frequently preferred augmentation materials.^[3,13–15]^ In the literature, there are still uncertainties regarding the augmentation methods and bone fixation of these grafts because there is no accepted treatment algorithm due to the fact that most of the published studies are case reports or the number of cases is very small and the results are inconsistent.^[16–18]^ The aim of the treatment is to correct the shortening in the extensor mechanism, obtain adequate fixation with appropriate tension and ensure tissue healing in a short time.^[5,19,20]^

The aim of this study is to examine the results of double-row reconstruction with autologous hamstring tendon graft in patients who developed PTR after TKA We hypothesize that this novel repair technique is a powerful reconstruction method that allows early mobilization without any additional surgical intervention and provides a return to activities similar to preinjury activities.

## 2. Materials and methods

Approval of the local ethics committee was obtained before data collection. Written informed consent was obtained from each patient. Fourteen patients with PTR who had surgical treatment with a double-row repair method using the hamstring tendons, at least 2 years of follow-up after surgery between 2006 and 2020, and whose complete records were available were included in the study. Inclusion criteria were determined as patients who underwent TKA and were subsequently diagnosed with PTR by clinical and radiological imaging methods. Exclusion criteria were determined as predisposing factors that will contribute to the development of PTR (rheumatological diseases, chronic renal failure, uncontrolled diabetes, and long-term steroid use), periprosthetic joint infection in the affected side knee joint according to the American Academy of Orthopedic Surgeons clinical practices guideline, patients whose interval between PTR diagnosis and surgery is longer than 6 months.

### 2.1. Preoperative evaluation

**Figure 1.**
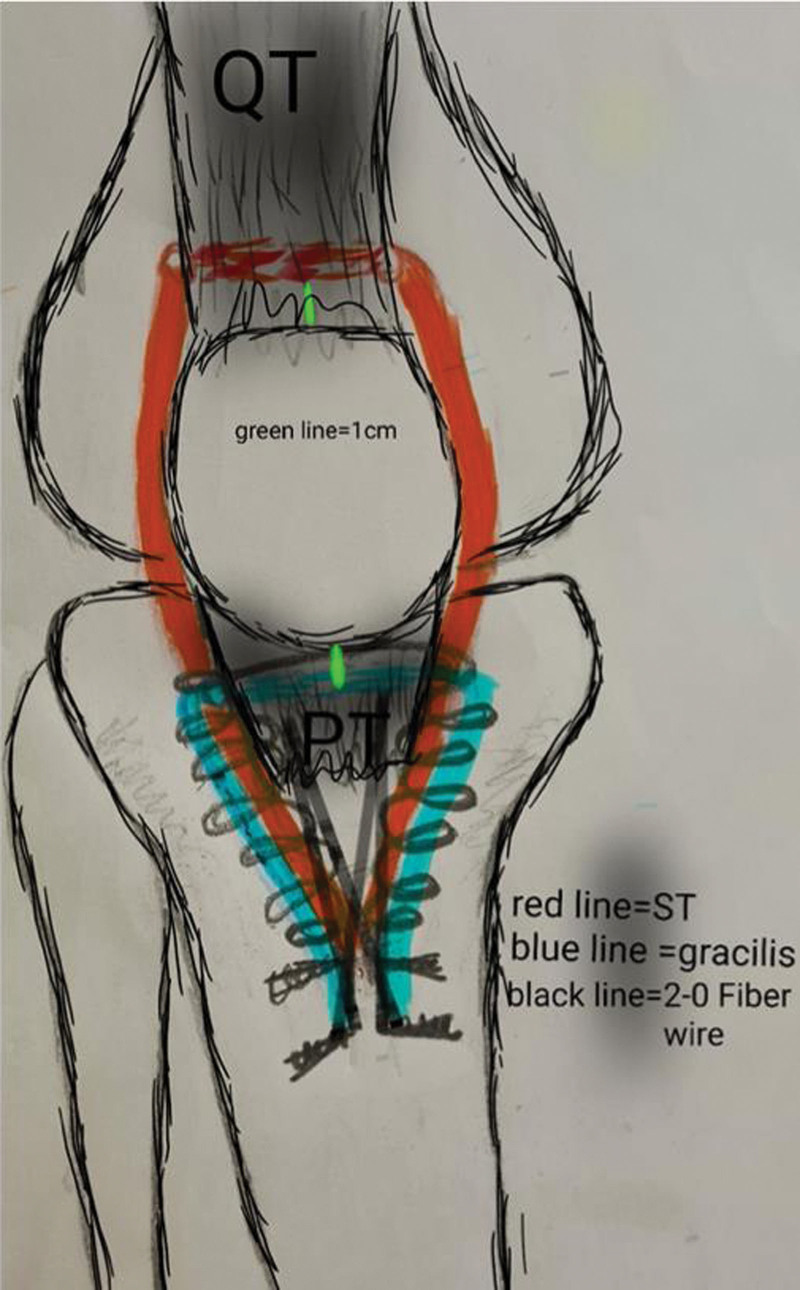
Schematic representation of the surgical technique.

Clinical and radiological evaluations of the patients were made by a senior orthopedist at the time of initial admission and at their last follow-up. The presence of gap and tenderness on the patellar tendon was examined in all patients. Active and passive knee joint range of motion was measured with a goniometer. The Caton-Deschamps index (CDI) is derived from lateral radiographic images of the knee and is calculated by dividing the distance between the lower pole of the patella and the anterior edge of the tibial plateau by the length of the patellar articular surface. The CDI was calculated on the plain radiograph taken at 30° flexion. In addition, in order to perform the double-row reconstruction technique, the distance between the proximal border of the patella and the upper edge of the tuberosity of the tibia was measured form the AP view. The closest length between the superior pole of the patella and the tuberosity of the tibia was important. Because during surgical repair, it was necessary to correlate the length of the semitendinosus (ST) autograft required for proximal row repair (Fig. 1). Patients also had USG and MRI imaging methods performed before the operation. In this way, retraction, fibrosis and atrophy in the extensor mechanism were evaluated. The degree of extension loss, Tegner-Lysholm knee score, and Kujala score were used to compare pre- and postoperative functional results.

The Tegner-Lysholm Knee Scoring System is a widely used clinical assessment tool designed to evaluate the functional status and subjective symptoms of individuals with knee injuries or conditions. The Tegner-Lysholm knee score comprises 8 items, each assigned a specific score based on the patient’s reported symptoms and functional limitations. Score range from 0 to 100, with higher scores indicating better knee function and fewer symptoms. A score of 100 represents normal knee function without any symptoms or limitations, while lower scores reflect increasing levels of impairment and disability.

The Kujala Score, also known as the Kujala Anterior Knee Pain Scale, is a clinical assessment tool designed to evaluate the severity of anterior knee pain and associated functional limitations. The Kujala Score consists of 13 items that assess various aspects of knee function, including pain, swelling, instability, and limitations in activities of daily living and sports participation. Scores on the Kujala Score range from 0 to 100, with higher scores indicating better knee function and fewer symptoms. A score of 100 represents normal knee function without any symptoms or limitations, while lower scores reflect increasing levels of impairment and disability.

### 2.2. Surgical technique

The patients were placed in the supine position with the affected extremity covered in a sterile manner by applying a tourniquet. A skin incision was made from the upper pole of the patella to 2 cm distal to the tibial tubercle using the old incision line. Dissection was performed until the rupture area was seen. Fibrotic and nonliving tissues in the area were resected and the living tendon end was exposed. By loosening the proximal adhesions, distal mobilization of the patella and patellar tendon was achieved. At this stage, it should be seen that the patella and patellar tendon can be mobilized more distally by approximately 2 cm. ST and gracilis tendons were identified through the same incision, removed from the tibial attachment site, and the proximal part was released with the help of a tendon scraper. At this stage, measuring the length of the ST graft is critical. It is important that it is longer than 22 cm for the continuation of the procedure. The shortest tendon length in our series was measured as 23 cm. This is sufficient for proximal row repair. The free ends of the grafts were suspended with number 2 Ultra-reinforced Fiber Wire suture in accordance with the Krackow technique. The patellar tendon was suspended from the torn distal end to the proximal with 2 number 2 Fiber Wire sutures.

**Figure 2.**
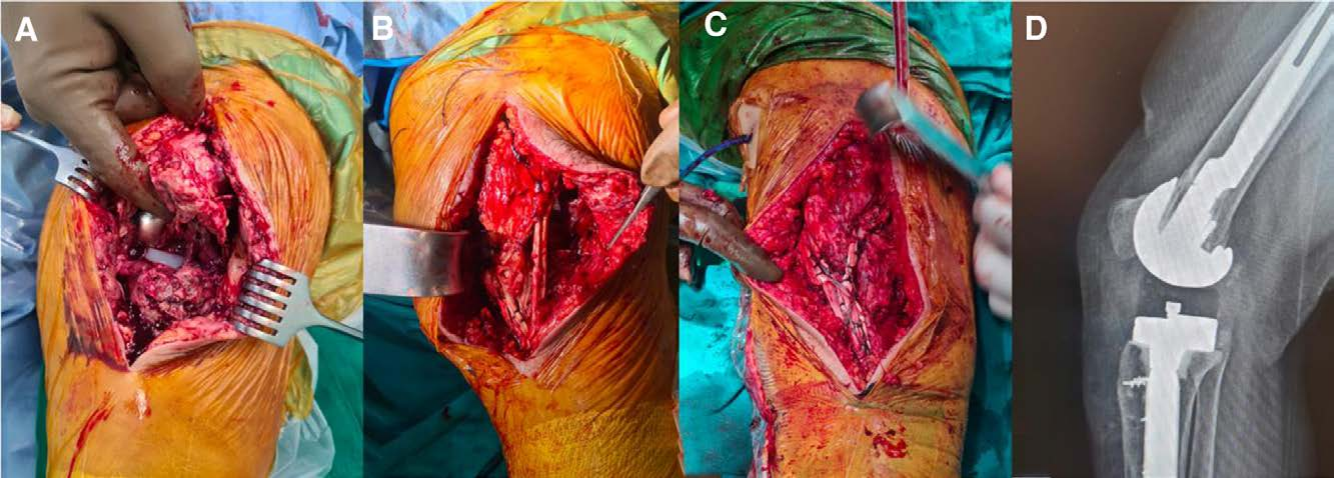
Intraoperative images of the patient. (A) Intraoperative patellar tendon tear image. (B) Image after the graft tendons are threaded and augmented. (C) Image after PEEK anchor placement and total reconstruction is completed. (D) Lateral view of postoperative plain radiograph.

While the ST graft is transferred through the quadriceps tendon by opening a transverse intratendinous tunnel 1 cm above the upper pole of the patella with a Kelly clamp, the same process is performed to transfer the gracilis graft through the patellar tendon 1 cm below the lower pole of the patella. Thus, double-row augmentation is completed proximal to the rupture. Augmentation is strengthened by connecting both grafts along the patella and patellar tendon, distally to the tuberosity of the tibia, to the remaining part of the adjacent patellar tendon with number 3 vicryl sutures. In this way, the patella and patellar tendon were augmented in a versatile way, including proximal, distal, medial and lateral, and the tension on the surface area was reduced (Fig. 2). Additionally, since no tunnel was opened to the patella, the risk of possible fracture was eliminated In the next step, the knee was flexed to 0° and all of the sutures in the ST graft and symmetrically half of the Krackow sutures in the patellar tendon were fixed to the medial and lateral sides of the proximal anatomical attachment site of the tuberosity of the tibia with 2 knotless polyetheretherketone (PEEK) anchors. Then, the sutures in the gracilis graft and the remaining Krackow sutures in the patellar tendon were fixed with 2 knotless PEEK anchors to the medial and lateral of the anatomical attachment site on the distal tuberosity of the tibia. Our aim here was to increase the contact surface between the graft and the bone and to provide a fixation that provides equal resistance against tension and torsional forces. After the reconstruction process was completed, the lateral and medial retinaculum were sutured to the augmented patellar tendon and grafts with no. 2 vicryl sutures (Fig. 2).

### 2.3. Postoperative rehabilitation

Postoperatively, patients were fitted with a hinged range of motion knee brace. Heel slide exercises were started to allow 30° flexion in the first 3 postoperative weeks. Patients were allowed to bear full weight with the help of the brace and walker. After the third week, walker support was terminated by allowing 90° flexion. After 6th week, the brace was terminated and the patient was submitted to physical therapy program.

### 2.4. Statistical analysis

The data were analyzed by Statistical Package for the Social Sciences (SPSS) (Version 25.0, IBM Corp, Armonk, NY). The findings were expressed as frequency and percentages. The Shapiro–Wilk test was used to determine the parametric or nonparametric distribution of the variables. Numeric variables are presented as mean ± standard deviation. The categorical variables were compared with Fischer’s exact test between the groups. The numeric variables were compared using the Mann–Whitney *U* test because of the number of patients. Repeated variables were analyzed with the Wilcoxon-signed ranks test. The statistical significance value was set at *P* < .05.

## 3. Results

In a total of 14 weeks of case series, 10 patients developed TKA and 4 patients developed PTR after TKA revision surgery. The mean age of the patients was 68.1 years (range 59–76). PTR developed within an average of 6.6 weeks (±4.1) after the TKA procedure. The median time between PTR and reconstruction was found to be 6.6 weeks (±4.1). Demographic and clinical data of the patients included in the study are given in Table 1.

**Table 1:** Demographic and clinical data of the patients.

	N (%)	Mean ± standard deviation
Age		68.1 ± 5.1
Gender		
Female	8 (57.1)	
Male	6 (42.9)	
Type of injury		
Spontaneous	7 (50.0)	
Simple falling	4 (28.6)	
Rehabilitation	3 (21.4)	
Rupture site		
Proximal	–	
Middle	3 (21.4)	
Distal	11 (78.6)	
Type of surgery		
Primary repair	10 (71.4)	
Revision	4 (28.6)	
The interval between injury and surgery (weeks)		6.6 ± 4.1

Passive joint range of motion was normal in all patients. The average active flexion range in the affected knee was 100° (range 80°–120°, SD = 12°), and the average active extension loss was 25° (range 10°–40°, SD = 10.3°). The mean CDI in the patients’ lateral radiographs taken at 30° flexion was found to be 1.8 (range 1.5–2.1, SD = 0.45). The average length between the upper pole of the patella and the tuberosity of the tibia was found to be 9.2 cm (range 8.2–12.4 cm, SD = 2.3).

Full-thickness PTR was detected in all patients. The tear was intratendinous in the distal region in eleven patients and was an avulsion tear in the tuberosity of the tibia in 3 patients. In the last follow-ups, the mean CDI was calculated as 1.25. When this value was compared to the preoperative value, there was a significant decrease (*P* = .014). The average extension loss decreased from 25° before surgery to an average of 5° (range 0°–15°; SD = 5.3°) postoperatively. The average postoperative flexion range of motion was found to be 100° (range 80°–120°; SD = 10.3°) at the last follow-up. The Tegner-Lysholm knee score increased from an average of 45 (range 35–53; SD = 10.1) preoperatively to an average of 77 (range 55.0–88; SD = 9.7) postoperatively (*P* < .01). The preoperative Kujala knee score increased from an average of 43 (range 35–69; SD = 4.5) to an average of 78 (range 56–85; SD = 7.6) after the surgery (*P* < .001). A statistically significant difference was found between both scores (Table 2).

**Table 2:** The Kujala, Tegner-Lysholm, and Caton-Deschamps index cores of the patients before and after surgery.

	Mean ± standard deviation		*P* value
	Preoperative assessment	Postoperative assessment	
Kujala patellofemoral score*	45.4 ± 4.5	75.9 ± 7.6	<0.001
Tegner-Lysholm knee scale score*	45.6 ± 10.1	75.9 ± 9.7	<.01
Caton-Deschamps index	16.7 ± 4.5	12.9 ± 1.6	.014

* Wilcoxon-signed ranks test.

Implant failure did not occur in any of the patients. No autograft or implant-related inflammation was encountered. Only 3 of our patients developed superficial skin infections in the early postoperative period, which was resolved with oral antibiotic treatment. Two patients had permanent hypesthesia on the lateral side of the knee in the postoperative period. No rerupture, patella fracture, or tuberosity tibia fracture was observed in any patient during follow-up. All but 2 patients stated that they could perform their daily activities. Both of these patients, who experienced activity deficiency, had a loss of extension of more than 10° and stated that they had difficulty especially in going up and down stairs. Both of these patients said that they could almost complete their daily activities with a cane. No additional patient required crutches at last follow-up.

## 4. Discussion

The main result of our study is reconstruction with autologous hamstring tendons can be safely applied with some modifications in the treatment of PTR occurring after TKA. We did not apply cast or immobilization to any of our patients. As a precaution, we recommended an angle-adjustable hinged knee brace, thus allowing postoperative mobilization and limited active-passive movements. It has already been shown in many studies that early movement is important in preventing postoperative complications such as joint movement limitation and quadriceps atrophy, and this was proven in our study. In previous studies, many dependent variables such as patient age, activity level, type of tear, location, time of occurrence, and underlying predisposing factors were considered and many different treatment methods were tried. There are still difficulties in treatment due to the lack of randomized studies on the management of PTR, and the insufficient number of patients in retrospective studies. Although PTR after TKA is a very rare event, it can result in serious complications if left untreated. In previous studies, the rate of patients affected after TKA was found to be between 0.17% and 2.5%.^[1–3,21,22]^

The time the tear occurs is important in managing of PTRs that develop after TKA. Tears that occurred less than 6 weeks are considered acute and the general consensus is that they can be repaired primarily.^[1,23,24]^ The biggest disadvantage of primary repair is that it requires long-term immobilization, so persistent pain in the knee, loss of flexion, patella infera and quadriceps muscle weakness are common complications after treatment.^[5,6]^ In patients in whom early mobilization is initiated to minimize these complications, the rate of repair failure increases and loss of active extension with patella alta develops.^[21]^ Many studies in the literature have reported rerupture or gap formation as a result of primary repair.^[25–28]^ However, most of these studies are in the form of case reports. Considering TKA patients, the rate of encountering these complications is even higher because reasons such as the advanced age of these patients, the presence of additional diseases, poor tissue quality, and wound healing problems reduce the adequacy of the primary repair in these patients. Li et al treated their patients who developed PTR after TKA with the primary repair method and encountered a failure rate of 26% and an average loss of extension of 21°.^[29]^ A similar situation was reported in the study of Courtney et al, who treated 58 patients who developed extensor mechanism failure, including PTR, with the primary repair method after TKA and encountered a failure rate of 44%. They also reported that the highest failure rate in the study was in PTR.^[24]^ Seven of the patients in our study were classified as acute tears and the remaining 7 as chronic tears, and the primary repair method was not tried in any of our patients before reconstruction.

Previous studies on allografts have been described in the literature. Acceptable results were obtained in some of them, but overall there seems to be a lot of inconsistency between studies. Generally, tendon allografts play an important role, especially in cases where there is a large local tissue deficiency. It is widely used in ligament and tendon reconstructions, especially around the knee.^[30–34]^ Its most important advantages include the absence of donor site morbidity, high tensile strength, reduced surgical time and resulting in lower arthrofibrosis. Disadvantages include limited availability, high cost, risk of rejection due to immune incompatibility between donor and recipient, and risk of bacterial and viral infection transmission. In addition, animal studies with allografts have shown that revascularization, cell repopulation, and remodeling take much longer compared to autografts.^[35,36]^ This is reflected in clinical studies as a higher rate of rerupture and the need for longer immobilization. Lewullis et al^[37]^ reported that out of 8 patients they reconstructed with allografts after TKA, 2 had early infection, 6 had no functional recovery, and knee function scores worsened over time. In another large-scale study, Nazarian and Booth^[38]^ used allografts in 36 patients who developed extensor mechanism failure, including PTR, after TKA, and found rerupture in 8 patients. The results of the reconstruction method performed by Leopold et al with allografts containing the entire extensor mechanism were quite dramatic. They used allografts in 7 knees of 6 patients and failed in all of them. They found that all but 1 patient was dependent on assistive walking devices and had to revise 4 patients with different surgical methods.^[20]^ In another similar study, Diaz-Ledezma et al^[39]^ performed reconstruction with Achilles tendon allograft in 29 knees of 27 patients with PTR after TKA and reported unsuccessful results in eleven cases, and reported that 8 of these cases failed due to infection.

As concerns about allografts have increased in recent years, alternative methods have come to the fore. The most current of these are materials known as synthetic grafts (Dacron tape, Marlex mesh, etc). These synthetic materials have been used in orthopedic oncology surgery for a long time. Compared to allografts, their biggest advantages are their ubiquity, lower cost, no risk of disease transmission, and unlimited usability. Moreover, they biologically serve as a framework for the proliferation of fibroblasts and biomechanically, unlike allografts, they do not stretch over time.^[40,41]^ There are also several studies published in recent years regarding the use of synthetic grafts in PTR after TKA. In their study of 4 cases, Hasegawa et al^[42]^ used a high molecular weight polyethylene cable as a synthetic graft and encountered an infection in 1 patient that required removal of the graft in the third week after surgery, but they achieved successful results in all patients at their last follow-up. In Wood et al’s series of 27 patients, they reconstructed 13 of the patients who developed extensor mechanism failure after TKA with a synthetic graft and 14 with an allograft and published their results. According to the study, none of the patients in the synthetic graft applied group developed graft failure, and 15% of the patients had an infection that required removal of the graft. In the group where allograft was applied, the rate of graft failure was found to be 21%, and the rate of infection requiring removal of the graft was found to be 21%. In addition, loss of extension in the group where the allograft was applied was significantly higher than in the group where the synthetic graft was applied.^[41]^

The high failure rates of allografts and the need to prove the reliability of synthetic grafts with more studies have made the use of autogenous autografts popular again in recent years, and different surgical techniques have been described. The most commonly preferred autogenous grafts are ST and gracilis tendons. Differently, autogenous tendon grafts with bone blocks and medial gastrocnemius muscle have also been rarely used in the repair of PTRs.^[43–45]^ However, there are very few studies on these in the literature. The medial gastrocnemius muscle is widely used especially in tumor surgery. Although it is applied in PTRs to fill large gaps, wound closure problems are frequently encountered due to its high volume. In addition, its disadvantages are that it degenerates over time and causes loss of knee movements.^[43]^

The use of autogenous hamstring tendons in the reconstruction of PTRs was first described by Kelikian et al.^[46]^ Over time, it has been modified by making some differences in technique. The following are noteworthy as common features of most of the applied techniques: The aim is to preserve the distal connections of the tendons, thus ensuring sufficient blood flow to keep the tendon alive, to pass the grafts through the bone by opening transverse tunnels to the patella and tibia, and to perform single-row fixation both proximally and distally.^[47–49]^ Although good results have been reported with these techniques, complications have also been reported. Patella and proximal tibia fractures, lack of extension, and graft reruptures, especially due to tunnel use, are among these complications. Regardless of these techniques, we did not use bone tunnel for tendon fixation in the patella and tibia. By taking advantage of the length of the ST graft (>23 cm), by passing it through the intratendinous tunnel in the quadriceps tendon 1 cm proximal to the patella, and bypassing the gracilis tendon through the patellar tendon 1 cm distal to the patella, we augmented the patella both proximally and distally, increased the tendon-tendon contact surface and increased the tension strength during distal shifting and distributed it more evenly. We also reduced the risk of patella fractures that may develop. For the fixation of the graft to the tibia, we fixed the ST graft to the proximal tuberosity of the tibia and the gracilis graft to the distal part of the tibia in full extension, taking into account the anatomical attachment point of the patellar tendon with the help of a knotless PEEK anchor. Thus, we aimed to increase the healing potential and speed and achieve more anatomical healing by increasing the tendon-bone contact surface. We did not encounter any problems with tendon length in any of our patients. Again, none of our patients developed patella or proximal tibia fractures. Only 2 of our patients had more than 10 points of extension loss, and these patients were able to perform their daily activities with the help of a cane. Again, none of our patients developed implant failure, graft rerupture, or infection requiring surgery.

Our study has some limitations. Relatively small sample size is one of them, making statistical analysis of the data difficult. The lack of a control group prevented us from drawing definitive conclusions. However, we would like to point out that this injury is very rare. Additionally, not measuring quadriceps muscle strength is another weakness of the study.

In conclusion, the double-row repair method we perform with autologous hamstring tendons taken from the same side is safe, applicable, gives good results, and provides functional recovery in patients, especially in distal tears that do not exceed 6 months. It also allows patients to return to their preinjury daily activities. We think that our study will make important contributions to other researchers in the future in the development of a treatment algorithm for this injury.

## Acknowledgments

The authors thank to all hospital workers for their high efforts.

## Author contributions

**Conceptualization:** Erkan Akgun, Abdulsamet Emet, Emre Tepedelenlioglu, Kemal Sibar, Halil Gok, Ahmet Firat.

**Data curation:** Erkan Akgun, Abdulsamet Emet.

**Formal analysis:** Erkan Akgun, Emre Tepedelenlioglu, Kemal Sibar, Halil Gok, Ahmet Firat.

**Investigation:** Ahmet Firat.

**Methodology:** Erkan Akgun, Abdulsamet Emet, Emre Tepedelenlioglu, Kemal Sibar, Halil Gok, Ahmet Firat.

**Supervision:** Erkan Akgun, Abdulsamet Emet, Emre Tepedelenlioglu, Ahmet Firat.

**Visualization:** Kemal Sibar, Ahmet Firat.

**Writing – original draft:** Erkan Akgun, Abdulsamet Emet, Kemal Sibar, Halil Gok, Ahmet Firat.

**Writing – review & editing:** Abdulsamet Emet, Emre Tepedelenlioglu, Ahmet Firat.
